# Prescription Patterns of Antiseizure Medication in Adult Patients with Epilepsy in Kazakhstan (2021–2023)

**DOI:** 10.3390/medsci13040276

**Published:** 2025-11-19

**Authors:** Dina Kalinina, Temirgali Aimyshev, Alimzhan Muxunov, Zhassulan Utebekov, Gaziz Kyrgyzbay, Darkhan Kimadiev, Guldana Zhumabaeva, Abduzhappar Gaipov, Antonio Sarria-Santamera

**Affiliations:** 1 Department of Medicine, School of Medicine, Nazarbayev University, Astana Z05P3Y4, Kazakhstan; temirgali.aimyshev@nu.edu.kz (T.A.); alimzhan.muxunov@nu.edu.kz (A.M.); abduzhappar.gaipov@nu.edu.kz (A.G.); antonio.sarria@nu.edu.kz (A.S.-S.); 2 Epileptology Centre, RSE Medical Centre Hospital of the President’s Affairs Administration of the Republic of Kazakhstan, Astana Z05M4E8, Kazakhstan; jase8522@gmail.com (Z.U.); doctorgaziz@gmail.com (G.K.); kimadiev96@gmail.com (D.K.); dana922012@gmail.com (G.Z.)

**Keywords:** epilepsy, anticonvulsants, drug prescriptions, drug therapy, combination, comorbidity

## Abstract

**Background/Objectives**: Epilepsy is a major neurological disorder associated with significant comorbidity and treatment challenges. In low- and middle-income countries, access to newer antiseizure medications (ASMs) remains limited, and prescription patterns often rely on older agents. This study aimed to characterize national prescribing patterns of ASMs among patients with epilepsy in Kazakhstan from 2021 to 2023. **Methods**: We conducted a retrospective observational study using de-identified electronic health record data from the Unified National Electronic Health System of Kazakhstan. All patients with an ICD-10 diagnosis of epilepsy (G40) and at least one ASM prescription during 2021–2023 were included. Prescription frequencies, therapy type, and chronic polytherapy levels were analyzed. Associations between therapy type, age, and comorbidity status were determined. **Results**: A total of 54,274 patients were identified (median age 42 years; interquartile range (IQR) 31–57). Monotherapy predominated: 61.7% remained on monotherapy, 18.5% remained on polytherapy, and 19.8% had mixed exposure. Carbamazepine and valproic acid were most frequently prescribed (64.3% and 45.6% of patients, respectively). Among those with chronic medication data (*n* = 15,752), nervous-system drugs were common (70.1%), led by psycholeptics (49.7%); frequently dispensed agents included chlorpromazine (*n* = 5991), clozapine (*n* = 1875), and risperidone (*n* = 1642). Cardiovascular agents were recorded in 37.2% (acetylsalicylic acid *n* = 4056; atorvastatin *n* = 2235), and diabetes drugs in 12.1% (metformin *n* = 1430). **Conclusions**: Epilepsy treatment in Kazakhstan remains dominated by older broad-spectrum ASMs, while the use of lamotrigine and levetiracetam is steadily increasing. The frequent co-prescription of psychotropic and cardiometabolic drugs underscores the need for coordinated, multidisciplinary care and continued monitoring of prescribing patterns to enhance treatment safety and effectiveness.

## 1. Introduction

Epilepsy affects about 1% of the global population, with nearly 80% of patients living in low- and middle-income countries (LMICs) where access to care remains limited [1]. In Central Asia, the burden of epilepsy is increasing; in Kazakhstan, incidence and prevalence rose from 26.1 and 73.1 per 100,000 in 2014 to 88.8 and 73.1 per 100,000 in 2020, respectively [2,3]. Despite this trend, up to three-quarters of people with epilepsy in LMICs remain untreated, compared with about 10% in high-income countries [4]. Limited drug availability, shortages of trained specialists, and social stigma contribute to this persistent treatment gap [5,6]. Improving access to effective and affordable antiseizure medications (ASMs) through national health programs is therefore a major priority in these regions.

According to current guidelines, the optimal management of epilepsy relies on ASMs to prevent recurrent seizures [7]. Monotherapy is typically preferred as the first-line treatment because approximately 60–70% of patients can achieve long-term seizure remission with a single ASM [8,9,10]. Clinical guidelines recommend monotherapy whenever possible due to lower risks of adverse effects and drug interactions [11,12,13]. Polytherapy, defined as the prescription of two or more ASMs concurrently, is usually reserved for refractory epilepsy cases where seizures are not controlled by one medication or in specific scenarios such as multiple seizure types [14]. Balancing efficacy and side effects is crucial; while polytherapy may improve seizure control in difficult cases, it also increases treatment complexity and the risk of toxicity [15].

ASM prescription patterns have evolved over time with the introduction of newer medications. Traditional “first-generation” ASMs, such as valproic acid and carbamazepine, remain widely used, especially in LMICs, due to their broad effectiveness and familiarity [16,17,18,19]. Over the past two decades, newer ASMs (e.g., levetiracetam, lamotrigine, and topiramate) have gained popularity due to their improved safety profiles and specific indications [20,21,22]. Monitoring trends in the use of ASMs is important to ensure alignment with current evidence and guidelines [23]. For example, concerns about valproate’s teratogenicity have prompted initiatives to limit its use in women of childbearing age [24,25]. Changes in prescribing trends can also reflect the dissemination of new guidelines, the availability of new drugs, and the influence of healthcare policies [26].

Comorbidities represent another critical aspect of epilepsy care. Patients with epilepsy often have co-occurring psychiatric and somatic conditions. Approximately one-third of individuals with epilepsy have at least one psychiatric disorder, such as depression or anxiety, which is several-fold higher than that of the general population [27,28,29]. Epidemiologic data also show elevated rates of cardiovascular and metabolic conditions among patients with epilepsy [30,31,32,33]. These comorbidities can complicate treatment: clinicians must consider drug–drug interactions and, when possible, choose ASMs that also benefit or at least do not worsen comorbid conditions [34,35]. Conversely, the presence of multiple comorbidities often leads to polypharmacy, increasing the risk of side effects and adherence challenges [36].

In this study, we analyzed prescription data from 2021 to 2023 to characterize current ASM prescribing patterns and treatment strategies among patients with epilepsy in Kazakhstan. We provide a comprehensive overview of the distribution of ASMs and their combinations, the relative use of monotherapy versus polytherapy and the prevalence of comorbid conditions together with associated non-ASMs. Variations by epilepsy subtype (ICD-10 codes) and region were also explored. This study aims to identify gaps in care and opportunities for improving epilepsy management by benchmarking these findings against prior data and international norms. Ultimately, this research is intended to inform clinicians, policymakers, and public health officials working to optimize therapy for epilepsy—a condition that imposes a significant burden not only through seizures but also through its frequent comorbidities and psychosocial implications.

## 2. Materials and Methods

### 2.1. Study Population and Data Sources

We conducted a retrospective observational study using de-identified electronic health record data from the Unified National Electronic Health System of Kazakhstan for the years 2021–2023. This database captures outpatient prescription dispensations and associated diagnoses across all regions of the country. We included adults (≥18 years) with a documented diagnosis of epilepsy (ICD-10 code G40 or any G40.x subgroup), who received at least one ASM prescription during the study period. Patients under 18 years were excluded. The final cohort included 54,274 unique patients.

The study was conducted in accordance with the Declaration of Helsinki and approved by the Local Bioethics Committee of the Hospital (Protocol No. 4; 20 December 2024). Informed consent was waived due to the retrospective analysis of anonymized, de-identified data.

### 2.2. Definitions

Patients were considered present in a given calendar year if they had at least one ASM dispensed in that year. Cohort entry was defined as the first observed year and exit as the absence of subsequent dispensations, allowing dynamic participation across 2021–2023. Each dispensation event was counted as a prescription for descriptive totals. At cohort entry, we tabulated ICD-10 G40.x categories and summarized them for descriptive purposes. All ASMs were identified and summarized to capture patient-level prevalence and prescription counts. All non-ASM comedications were mapped to WHO ATC main anatomical groups. For these non-ASM drugs, we reported the proportion of patients with at least one prescription in each group and highlighted commonly used classes and subclasses, as well as leading individual agents to illustrate practice patterns. Therapy was classified as monotherapy when only one ASM was active at a time and as polytherapy when exposure periods for two or more ASMs overlapped. Sequential single-agent changes without overlap were still considered monotherapy. Patients were categorized as always monotherapy, always polytherapy, or mixed if both occurred during 2021–2023. Transitions between years (2021 → 2022 and 2022 → 2023) were labeled as intensified (from mono- to polytherapy), deintensified (from poly- to monotherapy), switched (both directions), or same. In a predefined chronic-polypharmacy subset (*n* = 15,752) with complete chronic-use metadata, we enumerated concurrent drug counts by category (ASMs, somatic, psychiatric). The same overlap logic was applied to construct distributions (2, 3, 4, …, ≥10 agents) across categories. For regional analyses, patients were assigned to one of 17 administrative regions or to one of three major cities (Almaty, Astana, Shymkent) based on residence recorded in the national system. We compared therapy patterns (monotherapy share), age, and comedication burden between the pooled major-city group and other regions.

### 2.3. Statistical Analysis

Continuous variables were summarized as median (interquartile range (IQR)), and categorical variables as counts (percentages). Group comparisons were performed using Pearson’s χ^2^ test for categorical variables and the Wilcoxon rank-sum test for continuous variables. For year-to-year descriptive trends, we did not test across calendar years because of within-person correlation; regression analyses were conducted at the patient-level. Factors associated with escalation to polytherapy were examined with a multivariable logistic regression estimating the odds of switching from monotherapy to polytherapy at any time during 2021–2023. Covariates included age (per 5-year increment), comorbidity burden (count of distinct chronic non-ASM drugs per patient), and ICD-10 category at the first ASM (reference: G40.8). We reported odds ratios with 95% confidence intervals and two-sided *p*-values; *p* < 0.05 was considered significant. All analyses were performed in R (version 4.3.0) using the following packages: dplyr, tidyr, stringr, purrr, forcats, lubridate, ggplot2, scales, broom, and knitr, (with base stats functions for χ^2^, Wilcoxon tests, Fisher’s test, and logistic regression).

## 3. Results

### 3.1. Patient Characteristics

A total of 54,274 patients with epilepsy were identified between 2021 and 2023. The median age was 42 years (IQR 31–57, range 18–99). The most frequently recorded ICD-10 codes were G40.8 “other epilepsy” (24.9%), G40 “epilepsy” not otherwise specified (21.4%), G40.2 localization-related symptomatic with complex partial seizures (20.6%), and G40.3 generalized idiopathic epilepsies (19.6). Less frequent codes were G40.1 simple partial (10.2%), G40.4 other generalized (6.3%), and G40.9 unspecified (8.1%); G40.5–G40.7 were rare (each ≤ 0.8%). Because more than one code could be assigned over time, categories were not mutually exclusive (Table 1). Regarding treatment levels across observed years, 61.7% were consistently on monotherapy (*n* = 33,471), 18.5% consistently on polytherapy (*n* = 10,052), and 19.8% had mixed exposure (*n* = 10,751). At least one prescription for the following ASMs was recorded: carbamazepine in 64.3% (*n* = 34,894), valproic acid 45.6% (*n* = 24,766), lamotrigine 20.4% (*n* = 11,070), levetiracetam 15.2% (*n* = 8263), topiramate 8.8% (*n* = 4794), and oxcarbazepine 2.4% (*n* = 1327).

### 3.2. ASM Prescription Patterns

**Figure 1:**
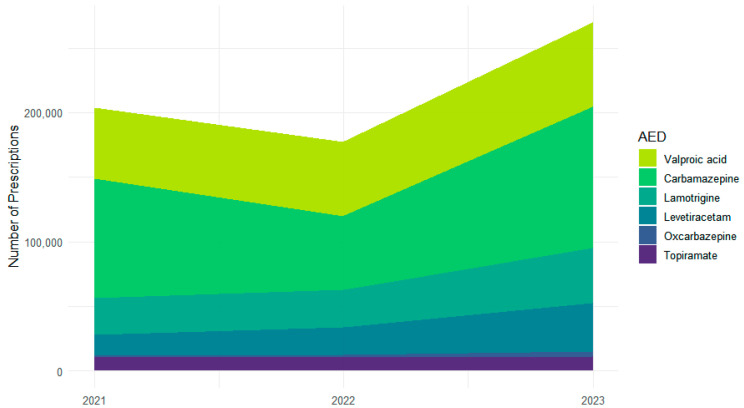
Changes in individual ASM prescription volume across 2021–2023.

Across 2021–2023, the six most frequently used ASMs accounted for 651,377 prescriptions: carbamazepine (*n* = 259,202), valproic acid (*n* = 177,605), lamotrigine (*n* = 100,180), levetiracetam (*n* = 74,455), topiramate (*n* = 33,023), and oxcarbazepine (*n* = 6912) (Figure 1). Year-specific totals were characterized by a dip and subsequent increase for carbamazepine (92,607 in 2021; 57,023 in 2022; 109,572 in 2023) and steady increases for valproic acid (54,531; 57,594; 65,480), lamotrigine (from 28,147 to 29,327 to 42,706), and levetiracetam (from 16,636 to 20,847 to 36,972). Topiramate remained stable (10,822; 10,645; 11,556). Oxcarbazepine increased from a low baseline (872; 2158; 3882).

**Figure 2:**
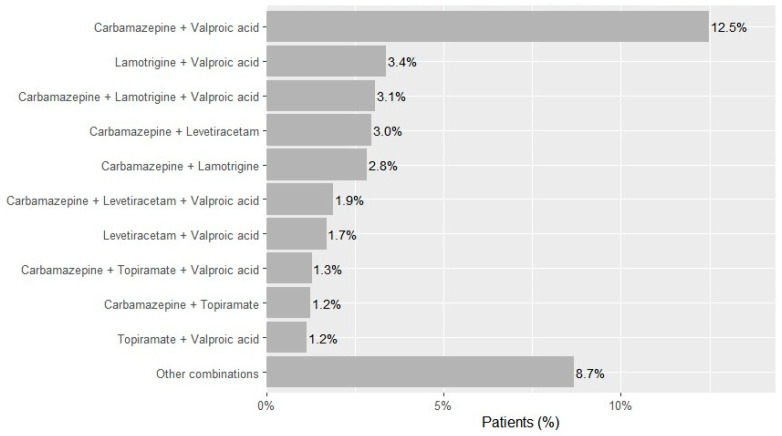
Most frequent ASM combinations in patients with epilepsy in Kazakhstan across 2021–2023.

Two-drug regimens accounted for 68.9% of observed combinations (*n* = 15,214), most frequently carbamazepine and valproic acid (*n* = 6773; 12.5% of the cohort), lamotrigine and valproic acid (*n* = 1834; 3.4%), carbamazepine and levetiracetam (*n* = 1612; 3.0%), carbamazepine and lamotrigine (*n* = 1542; 2.8%), levetiracetam and valproic acid (*n* = 924; 1.7%), carbamazepine and topiramate (*n* = 676; 1.2%), and topiramate and valproic acid (*n* = 627; 1.2%) (Figure 2). Three-drug regimens accounted for 23.7% of combinations (*n* = 5244)—dominated by carbamazepine-lamotrigine-valproic acid (*n* = 1666; 3.1% of the cohort), carbamazepine-levetiracetam-valproic acid (*n* = 1027; 1.9%), and carbamazepine-topiramate-valproic acid (*n* = 700; 1.3%). Regimens with ≥4 drugs were uncommon overall (four drugs *n* = 1397; five *n* = 228; six *n* = 7; ~3.0% of the cohort).

**Figure 3:**
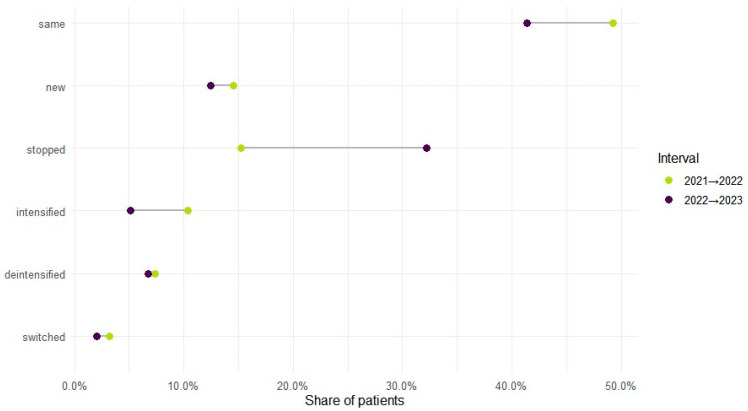
ASM treatment regimen changes across years.

Therapy level varied by year: monotherapy accounted for 70.1% of regimens in 2021, 65.7% in 2022, and 72.4% in 2023, with polytherapy showing the reciprocal pattern (29.9%, 34.3%, 27.6%). Across adjacent years, intensification (mono to poly) occurred in 10.4% from 2021 to 2022 and 5.2% from 2022 to 2023; de-intensification (poly to mono) in 7.4% and 6.8%; and bidirectional switching in 3.2% and 2.1%, respectively. In total, 71.7% (*n* = 38,924) maintained the same therapy level during observed years, while 28.3% (*n* = 15,350) changed at least once (Figure 3). Cohort entry and exit were dynamic: 41,364 entered in 2021; 7904 were new in 2022 (14.6% of the cohort) and 5006 in 2023 (9.2%); 8279 ceased to appear in 2022 (15.3%) and 17,464 in 2023 (32.2%).

### 3.3. Mono-to-Polytherapy Transitions Among ASM-Treated Patients

Overall, 6664 of 54,274 patients (12.3%) were observed to escalate from monotherapy to polytherapy during 2021–2023. Median age at first ASM was similar in non-switchers versus switchers (39 [28–54] versus 39 [29–52]; *p* < 0.01), yet an inverse association with age was estimated in the adjusted model: OR 0.98 (95% CI 0.98–0.99) per 5 years; *p* < 0.01. Comorbidity burden (distinct non-ASM chronic drugs per patient) was not associated with switching: OR 1.00 (0.98–1.01); *p* = 0.82. Using G40.8 as the reference ICD code at the first ASM prescription, higher odds of switching were observed for G40.3: OR 1.15 (1.06–1.24); *p* < 0.01. Lower odds were found for G40.2: OR 0.85 (0.78–0.92); *p* < 0.01 and for G40.1: OR 0.89 (0.81–0.99); *p* = 0.03. Other categories showed no significant associations (Table 2).

### 3.4. Chronic Polytherapy Patterns

Among patients with chronic medication data (*n* = 15,752), treatment intensity was skewed toward lower counts (Table 3). For ASMs, two-drug regimens were most frequent (26.6%), followed by three-drug courses (10.2%); higher ASM counts were rare. Somatic and psychiatric drug classes showed longer right tails: while two to three agents predominated, a minority received five or more. Considering all chronic drugs, most patients used two to four agents (two drugs 22.4%, three drugs 13.3%, four drugs 8.4%), whereas only small fractions used nine or more. Co-prescribing was common: 62.9% received both ASMs and somatic drugs (*n* = 9909), 50.8% received both ASMs and psychiatric drugs (*n* = 8000), and 13.9% received agents from all three classes (*n* = 2197).

By ATC, 70.1% received nervous-system drugs (N), led by psycholeptics (N05, 49.7%). Frequently dispensed agents included chlorpromazine (*n* = 5991), clozapine (*n* = 1875), levomepromazine (*n* = 1831), risperidone (*n* = 1642), trihexyphenidyl (*n* = 1704), and amitriptyline (*n* = 1259). Cardiovascular drugs were used by 37.2% (notably lipid-modifying agents, 14.2%—atorvastatin *n* = 2235—and beta-blockers, 7.9%—bisoprolol *n* = 1159), and 27.8% received agents for blood and blood-forming organs (antithrombotics 27.5%—acetylsalicylic acid *n* = 4056). Alimentary and metabolism drugs were used by 15.1% (diabetes drugs 12.1%—metformin *n* = 1430). Other classes included endocrine (8.3%; thyroid therapy 6.6%), respiratory (4.9%), anti-infectives (4.1%), and antineoplastic/immunomodulating (4.0%) (Table 4).

### 3.5. Regional Patterns in Treatment and Comorbidity

**Figure 4:**
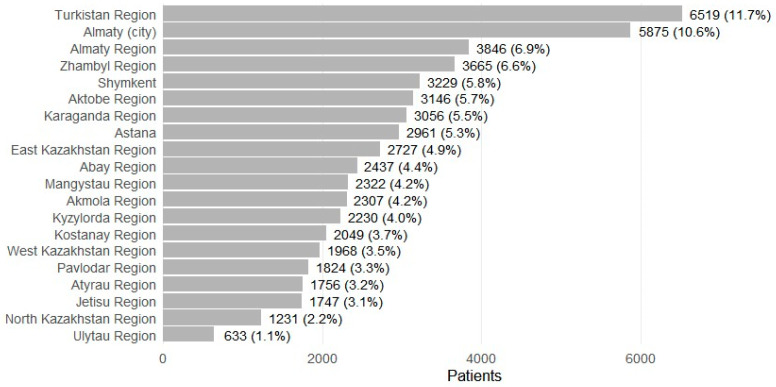
Patients with epilepsy receiving ASMs by regions (2021–2023).

Patients were recorded in 17 regions and 3 major cities (Figure 4). The largest shares were from Turkistan Region (*n* = 6519; 11.7%) and Almaty city (*n* = 5875; 10.6%), followed by Almaty Region (*n* = 3846; 6.9%), Zhambyl (*n* = 3665; 6.6%), Shymkent (*n* = 3229; 5.8%), Aktobe (*n* = 3146; 5.7%), Karaganda (*n* = 3056; 5.5%), and Astana (*n* = 2961; 5.3%). Smaller contributions included Ulytau (*n* = 633; 1.1%) and North Kazakhstan (*n* = 1231; 2.2%). Prescription volume broadly mirrored patient distributions, with higher absolute numbers in urban centers (for example, Almaty city 133,941 total prescriptions; Astana 53,344), and substantial ASM dispensing alongside comorbidity treatment in high-volume regions (for example, Karaganda 61,172 total; Aktobe 54,646; Almaty Region 56,489; Zhambyl 59,102). Regions with smaller populations had correspondingly fewer prescriptions (for example, Ulytau 5763; North Kazakhstan 13,178).

Across 2021–2023, 21.8% of patients resided in the three main cities (Almaty, Astana, Shymkent; *n* = 11,952) and 78.2% in other regions (*n* = 42,875). Monotherapy was more frequent in the main cities (63.1%) than in other regions (58.5%), while polytherapy was less common (36.9% versus 41.5%; both *p* < 0.01). Median age was slightly higher in cities (41 years [IQR 29–56]) than elsewhere (40 [29–54]; *p* < 0.01). Comorbidity burden was modestly higher in cities (median 2 [1–4]) versus other regions (2 [1–3]; *p* < 0.01) (Table 5).

## 4. Discussion

In this nationwide analysis of adult epilepsy patients in Kazakhstan (2021–2023), we observed that monotherapy remains the dominant treatment approach. These findings underscore that most patients can be maintained on one ASM, consistent with clinical guidelines recommending monotherapy whenever possible [37]. Notably, our monotherapy rate (61.7%) is higher than that reported in a multicenter study from India (42.6% monotherapy) [38], but lower than findings from a nation-wide Norwegian prescription study reporting 82% monotherapy [39]. A higher monotherapy share can reflect effective seizure control in routine care and prudent step-up to combinations only when needed, but may also mirror constraints in drug availability. Clinically, prioritizing single-agent regimens supports adherence and reduces adverse effects [40].

Carbamazepine and valproic acid dominated prescribing, with carbamazepine recorded for 64.3% and valproate for 45.6% of patients. This pattern resembles findings from several LMICs where older ASMs remain first-line and reflects Kazakhstan’s national reimbursement framework, in which these agents are long included in the formulary and widely available through the ambulatory drug supply system [41,42]. Their accessibility under state coverage likely sustains their predominant use, whereas the gradual rise in lamotrigine and levetiracetam reflects expanding public procurement and reimbursement of newer-generation ASMs with more favorable interaction profiles [43,44,45]. The transient dip in carbamazepine prescriptions—alongside a brief uptick in polytherapy—may indicate temporary supply or procurement delays in 2022 rather than a clinical preference shift. More recently introduced and higher-cost agents, such as oxcarbazepine, remain rarely prescribed, consistent with limited reimbursement and affordability constraints typical of resource-limited settings [46,47,48].

Therapy dynamics were common: nearly one-third of patients changed intensity at least once across adjacent years. In adjusted analyses, older age was associated with lower odds of escalation (OR 0.98 per year), while ICD-10 category at first ASM distinguished groups modestly (higher odds for G40.3; lower for G40.2 and G40.1). These signals are consistent with clinical heterogeneity in drug responsiveness and underscore the need for timely referral of patients with persistent seizures to specialist care for advanced pharmacologic options, surgical evaluation, or neuromodulation [49,50,51].

Polypharmacy extended beyond antiseizure therapy, reflecting the burden of somatic and psychiatric comorbidities. Among patients with chronic medication data, two to three concurrent drugs were typical, though a notable minority used five or more. Approximately 29% received treatment for at least one comorbidity, and these individuals were more likely to require multiple ASMs, consistent with the observation that epilepsy accompanied by comorbidities—particularly structural brain lesions or progressive neurological diseases—is more difficult to control [52,53,54]. Psycholeptics were common; chlorpromazine was the most frequently dispensed psychotropic, followed by clozapine. This pattern raises two main concerns. First, the continued use of sedating typical antipsychotics as sleep or behavioral agents—described in some post-Soviet settings—may inflate apparent rates of “psychosis” treatment and expose patients to avoidable adverse effects [55,56,57,58]. Second, clozapine’s dose-dependent seizure risk complicates co-management with ASMs, underscoring the need for close coordination between psychiatry and neurology [59,60,61,62]. Psychotropic medication use overall was high: nearly half of patients with comorbidities received antipsychotic or anxiolytic drugs, exceeding the expected prevalence of 20–30% for affective or anxiety disorders and only a few percent for psychosis [63,64,65]. One likely explanation is the off-label use of low-dose typical antipsychotics such as chlorpromazine or levomepromazine for sedation or behavioral control [52,53,54,55]. The prominence of chlorpromazine as the leading comedication warrants re-evaluation, given its adverse effect profile and the availability of safer alternatives [56,57,66]. The high use of trihexyphenidyl supports that many patients were treated with older antipsychotics and developed extrapyramidal symptoms [67,68].

Cardiovascular and metabolic drugs were prescribed to about one-third of comorbid patients, consistent with the older age distribution of epilepsy and the known overlap with hypertension, dyslipidemia, and diabetes [31,69,70,71]. Some ASMs require careful coordination with primary care: enzyme-inducing agents such as carbamazepine, phenytoin, and phenobarbital can raise lipid levels and reduce the effectiveness of anticoagulants, whereas valproate contributes to weight gain and insulin resistance [72,73,74,75,76,77,78,79,80]. The use of aspirin in roughly one-quarter of patients reflects the high burden of vascular disease and the role of stroke as a leading cause of late-onset epilepsy, highlighting the importance of integrating secondary prevention into epilepsy management [81,82]. Likewise, the frequent prescriptions of diabetes drugs (12.1%) and statins (14.2%) underscore the need to combine seizure control with cardiometabolic risk reduction and to consider the metabolic profiles of ASMs in treatment planning [31,75,80].

We identified noteworthy regional disparities in treatment patterns. Monotherapy rates were higher in the major urban centers (Almaty, Astana, Shymkent) compared to more peripheral regions, whereas the overall comorbidity burden appeared greater in city patients. These differences likely reflect variations in healthcare access and patient case-mix between urban and rural areas. Urban centers host specialized neurology services and experienced epileptologists, which may facilitate optimal management—physicians in tertiary centers might be more adept at achieving seizure control with a single well-chosen drug, and have access to a broader range of ASMs, than practitioners in rural areas. Our data suggest that big-city patients were more often kept on monotherapy, consistent with the presence of specialist care adhering to best practices. In contrast, patients from rural regions may have had higher rates of polytherapy (and possibly undertreated or uncontrolled epilepsy), which could be due to later referrals or limited drug availability locally. This pattern aligns with the known treatment gap in epilepsy care in low-resource settings [83,84]. Prior research in Southern Kazakhstan found the prevalence of epilepsy to be almost 60% higher in rural areas than urban areas (4.95 vs. 3.14 per 1000), which suggests that many rural patients historically did not receive optimal therapy or were not under active specialist follow-up [85]. Our finding that urban patients also carried a higher burden of comorbid illnesses (e.g., more on antithrombotics and statins in cities) could indicate that complex patients (such as elderly individuals with multiple conditions or those with stroke-related epilepsy) are preferentially managed in the city hospitals. Urban centers may thus see a concentration of both the most severe epilepsy cases and those with multiple health problems, leading to high comedication rates. This dual phenomenon—better adherence to monotherapy in cities, but also higher multimorbidity in city populations—highlights a challenge for healthcare planners. Efforts are needed to close the urban-rural gap by extending specialist training and telemedicine support to rural practitioners, ensuring that effective monotherapy is pursued whenever feasible even outside major hospitals. At the same time, resources in urban clinics must cater to the broader health needs of their patients, emphasizing multidisciplinary management (cardiology, endocrinology, psychiatry) alongside epilepsy care.

Our study’s strengths include a large, population-based sample and linkage of pharmacy dispensing with diagnosis codes and concomitant medications, allowing for broad clinical characterization [86]. However, certain limitations are inherent to administrative data and are typical of large-scale pharmacoepidemiologic studies. Gender information was incomplete in the data, preventing sex-based subgroup analysis. This limitation may have precluded evaluation of gender-related prescribing patterns. Clinical outcomes (such as seizure frequency, severity, or seizure freedom) and medication adherence cannot be directly inferred from dispensing records, as prescription data do not capture whether patients took medications as prescribed or the clinical rationale for therapy changes. Because clinical indications for switching from one ASM to another—such as inadequate seizure control, adverse effects, hepatotoxicity, or pregnancy considerations—are not recorded in administrative dispensing data, our study cannot determine the underlying reasons for treatment modifications. Overlapping days’ supply may occasionally misclassify short-term treatment switches as polytherapy, although this operational definition is standard in drug-utilization research [86,87,88,89]. Case identification based on ICD-10 codes may include some miscoded or single-seizure cases, but combining diagnostic codes with repeated ASM dispensations improves the positive predictive value [90,91,92]. Comorbidity estimates derived from medication data are conservative and may not distinguish multi-indication use [93,94]. These limitations are intrinsic to administrative population databases and do not compromise the validity of our findings.

In high-income health systems, use of carbamazepine and valproate has fallen while lamotrigine and levetiracetam have risen, as shown in national prescribing datasets [43,95]. By contrast, older, lower-cost agents remain prevalent in resource-constrained settings where availability and affordability shape treatment choices [96]. Ongoing surveillance of prescribing using individual-level dispensing data is therefore valuable for assessing concordance with evidence and detecting emerging signals [86]. Two examples illustrate the utility of such monitoring: (i) persistently high valproate use among women of childbearing potential should trigger targeted safety measures in light of dose-related teratogenic and neurodevelopmental risks [97,98], and (ii) a marked increase in polytherapy may reflect greater clinical complexity (i.e., more drug-resistant epilepsy) or suboptimal escalation in less severe cases, warranting audit against monotherapy-first principles [50].

## 5. Conclusions

In conclusion, this study provides a comprehensive overview of epilepsy management within a health system undergoing transition in the early 2020s. Concordance with established patterns—predominant monotherapy, continued use of older agents, and a substantial burden of comorbidities—supports the validity of the dataset and reinforces core principles of epilepsy care. At the same time, context-specific features—most notably the unusually frequent use of chlorpromazine—identify priorities for local audit and practice improvement. Sustaining robust seizure control while minimizing unnecessary polypharmacy and addressing broader health needs remains a central objective. Integrating care for psychiatric and medical comorbidities within epilepsy pathways is likely to enhance quality of life and may support better seizure outcomes, given the potential for conditions such as depression and diabetes to complicate management.

## Figures and Tables

**Table 1:** General characteristics of studied patients with epilepsy and current treatment of epilepsy (*n* = 54,274).

	Value
Age, median (IQR)	42 (31–57)
ICD, number of patients (%)	
G40	11,608 (21.4%)
G40.1	5509 (10.2%)
G40.2	11,170 (20.6%)
G40.3	10,632 (19.6%)
G40.4	3392 (6.3%)
G40.5	421 (0.8%)
G40.6	62 (0.1%)
G40.7	40 (0.07%)
G40.8	13,544 (24.9%)
G40.9	4408 (8.1%)
Level of therapy, number of patients (%)	
Monotherapy	33,471 (61.7%)
Polytherapy	10,052 (18.5%)
Switched from monotherapy to polytherapy	6664 (12.3%)
Switched from polytherapy to monotherapy	6135 (11.3%)
Prescribed ASMs, number of patients (%)	
Carbamazepine	34,894 (64.3%)
Valproic acid	24,766 (45.6%)
Lamotrigine	11,070 (20.4%)
Levetiracetam	8263 (15.2%)
Topiramate	4794 (8.8%)
Oxcarbazepine	1327 (2.4%)

**Table 2:** Factors associated with switching from monotherapy to polytherapy (*n* = 6664).

	OR (95% CI)	*p*-Value
Age	0.98 (0.98–0.99)	<0.01 *
Comorbid drugs	1.00 (0.97–1.01)	0.82
ICD		
G40.2	0.85 (0.78–0.92)	<0.01 *
G40.3	1.15 (1.06–1.24)	<0.01 *
G40.0	0.99 (0.91–1.08)	0.84
G40.1	0.89 (0.8–0.99)	0.03 *
G40.9	0.97 (0.87–1.08)	0.6
G40.4	1.00 (0.88–1.12)	0.94
G40	1.01 (0.87–1.16)	0.91
G40.5	0.71 (0.48–1.00)	0.06
G40.6	0.82 (0.31–1.75)	0.64
G40.7	0.85 (0.25–2.13)	0.75

Note: * = *p* < 0.05.

**Table 3:** Number of drugs used chronically by patients with epilepsy.

Chronic Polytherapy Level	ASMs	Somatic Drugs	Psychiatric Drugs	Total
2 drugs	4192 (26.6%)	2087 (13.2%)	1846 (11.7%)	3529 (22.4%)
3 drugs	1612 (10.2%)	1227 (7.8%)	974 (6.2%)	2095 (13.3%)
4 drugs	448 (2.8%)	796 (5.1%)	479 (3.0%)	1320 (8.4%)
5 drugs	73 (0.5%)	512 (3.3%)	309 (2.0%)	904 (5.7%)
6 drugs	2 (0.01%)	329 (2.1%)	140 (0.9%)	613 (3.9%)
7 drugs	0	222 (1.4%)	72 (0.5%)	389 (2.5%)
8 drugs	0	151 (1.0%)	25 (0.2%)	219 (1.4%)
9 drugs	0	106 (0.7%)	1 (0.006%)	146 (0.9%)
10 drugs	0	82 (0.5%)	0	97 (0.6%)
10+ drugs	0	129 (0.8%)	0	155 (1.0%)

**Table 4:** Medications used by the patients with epilepsy (*n* = 15,752) during 2021–2023 as categorized by the Anatomical Therapeutic Chemical (ATC) classification system.

Medications Classified According to ATC	*n* (%) of Patients
Alimentary tract and metabolism (A)	2373 (15.1%)
Drugs used in diabetes (A10)	1911 (12.1%)
Drugs for acid related disorders (A02)	345 (2.2%)
Antidiarrheals, intestinal anti-inflammatories/anti-infectives (A07)	53 (0.3%)
Laxatives (A06)	36 (0.2%)
Drugs for functional gastrointestinal disorders (A03)	22 (0.1%)
Digestives, incl. enzymes (A09)	5 (0.03%)
Other alimentary tract and metabolism products (A16)	1 (0.006%)
Blood and blood forming organs (B)	4371 (27.8%)
Antithrombotic agents (B01)	4332 (27.5%)
Antianemic preparations (B03)	24 (0.15%)
Antihemorrhagics (B02)	15 (0.1%)
Cardiovascular system (C)	5859 (37.2%)
Lipid modifying agents (C10)	2235 (14.2%)
Beta blocking agents (C07)	1247 (7.9%)
Diuretics (C03)	892 (5.7%)
Cardiac therapy (C01)	780 (4.9%)
Calcium channel blockers (C08)	390 (2.5%)
Agents acting on the renin-angiotensin system (C09)	315 (1.9%)
Genito-urinary system and sex hormones (G)	87 (0.5%)
Other gynecologicals (G02)	87 (0.5%)
Systemic hormonal preparations, excl. sex hormones and insulins (H)	1303 (8.3%)
Thyroid therapy (H03)	1032 (6.6%)
Corticosteroids for systemic use (H02)	260 (1.7%)
Pituitary and hypothalamic hormones and analogues (H01)	11 (0.07%)
Anti-infectives for systemic use (J)	648 (4.1%)
Antibacterials for systemic use (J01)	601 (3.8%)
Antivirals for systemic use (J05)	47 (0.3%)
Antineoplastic and immunomodulating agents (L)	633 (4.02%)
Antineoplastic agents (L01)	482 (3.1%)
Endocrine therapy (L02)	64 (0.4%)
Immunosuppressants (L04)	48 (0.3%)
Immunostimulants (L03)	39 (0.25%)
Musculo-skeletal system (M)	342 (2.2%)
Anti-inflammatory and antirheumatic products (M01)	303 (1.9%)
Drugs for treatment of bone diseases (M05)	34 (0.2%)
Muscle relaxants (M03)	5 (0.03%)
Nervous system (N)	11,044 (70.1%)
Psycholeptics (N05)	7831 (49.7%)
Anti-parkinson drugs (N04)	1713 (10.9%)
Psychoanaleptics (N06)	1259 (7.9%)
Analgesics (N02)	236 (1.5%)
Other nervous system drugs (N07)	5 (0.03%)
Antiparasitic products, insecticides and repellents (P)	29 (0.18%)
Antiprotozoals (P01)	29 (0.18%)
Respiratory system (R)	780 (4.9%)
Drugs for obstructive airway diseases (R03)	780 (4.9%)
Various (V)	2 (0.01%)
All other therapeutic products (V03)	2 (0.01%)

**Table 5:** Patient characteristics and ASM treatment profile in three main cities of Kazakhstan (Almaty, Astana, Shymkent) versus other regions, 2021–2023.

	Almaty, Astana, Shymkent	Regions	*p*-Value ^1^
Patients (*n*; % of cohort)	11,952 (21.8%)	42,875 (78.2%)	NA
Polytherapy share	36.9% (4410/11,952)	41.5% (17,792/42,875)	<0.01
Age, median [IQR] (years)	41 [29–56]	40 [29–54]	<0.01
Comorbid drugs per patient, median [IQR]	2 [1–4]	2 [1–3]	<0.01

^1^ Wilcoxon rank-sum; NA—not available

## Data Availability

The data that support the findings of this study are available from the Republican Center for Electronic Health of the Ministry of Health of the Republic of Kazakhstan. Restrictions apply to the availability of these data; they were used under license for the current study and are not publicly available. Data may be obtained from one of the authors (A. Gaipov) upon reasonable request and with permission of the Ministry of Health of the Republic of Kazakhstan.

## Abbreviations

The following abbreviations are used in this manuscript:

ASM(s)	Antiseizure medication(s)
ICD-10	International Classification of Diseases, 10th Revision
WHO ATC	World Health Organization Anatomical Therapeutic Chemical classification
LMICs	Low- and middle-income countries
LDL	Low-density lipoprotein
